# Chronic exertional compartment syndrome of the quadriceps femoris

**DOI:** 10.1016/j.radcr.2023.09.093

**Published:** 2023-10-21

**Authors:** David T. Ryan, Marion Hanley, Sarah K. Eustace, Stephen J. Eustace

**Affiliations:** a Department of Radiology, National Orthopaedic Hospital Cappagh, Dublin, Ireland; b Department of Radiology, University College, Dublin, Ireland

**Keywords:** Magnetic resonance imaging, Rectus femoris compartment syndrome, Selective fasciotomy

## Abstract

Chronic exertional compartment syndrome is well documented in the distal extremities, but is rare in the thighs. We present the case of a 19-year-old male who presented with chronic, recurrent bilateral thigh pain induced by physical activity, which settled with rest but recurred on immediate return to exercise. Postexercise MRI of both thighs demonstrated changes of symmetrical edema in the proximal quadriceps muscles, reflecting exercise-induce compartment syndrome. The patient underwent selective fasciotomies of each anterior thigh with improvement of symptoms. The patient is now doing well, with some residual milder symptoms and appearances on follow-up MR imaging are not as pronounced. This case describes the clinical presentation and imaging appearance of a rare case of chronic compartment syndrome in the quadriceps femoris.

## Introduction

Compartment syndrome can be acute (usually following direct trauma) or chronic (most commonly following exercise). While chronic exertional compartment syndrome is well described in many muscles of the upper and lower limb extremities, the phenomenon remains very rare in the thigh [1]. We present a rare case of exercise-induced compartment syndrome of the quadriceps muscles.

## Case report

A 19-year-old male who played amateur hurling, presented with a history of atraumatic, recurrent, bilateral thigh pain. He described the pain as a burning sensation with associated heaviness of his legs. This was induced by physical activity such as in hurling matches (a fast-paced amateur Irish field sport played with a ball and stick) or during intense self-directed training involving multiple squatting exercises. Symptoms usually settled with rest but tended to recur at a more rapid onset on immediate return to the same match or episode of physical activity/exercise. The history was overall more in keeping with chronic exertional compartment syndrome due its recurrent reproducible nature rather than an overuse injury. He had no prior medical history or history of anabolic steroid usage.

**Fig. 1:**
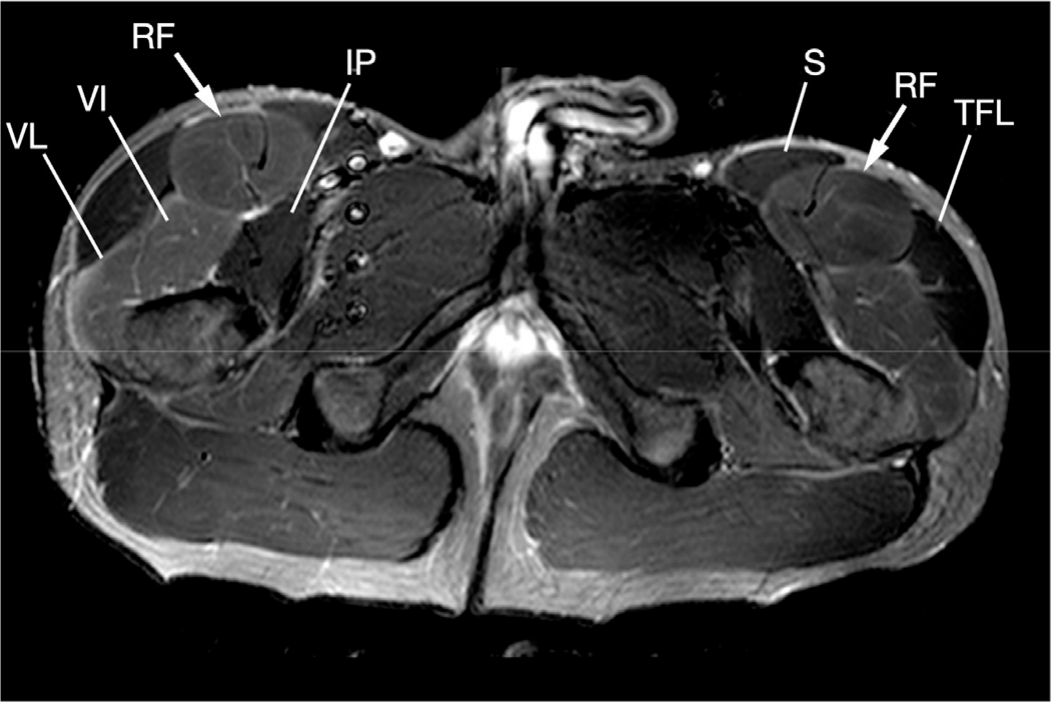
Axial STIR image of the proximal thighs demonstrates symmetrical muscular edema involving the bilateral rectus femoris (RF, arrowed) and vastus muscles (VI = vastus intermedius, VL = vastus lateralis), limited to the proximal thighs, with sparing of the hamstrings, adductors, sartorius (S) and tensor fascia lata (TFL) muscles bilaterally.

Intracompartmental pressure studies and a subsequent MRI were performed on the same day, after 30 minutes of exercise (when the patient began experiencing symptoms). Pressure studies were mildly elevated in both anterior thighs. Postexercise MRI of the thighs demonstrated symmetrical muscular edema involving the bilateral rectus femoris and vastus muscles, limited to the proximal thighs, with no muscle tear and sparing of the hamstrings, adductors, sartorius and tensor fascia lata muscles bilaterally (Fig. 1). This was performed using a 3T MRI (Achieva TX, Philips Healthcare) and a 16-element phased-array receive-only coil.

**Fig. 2:**
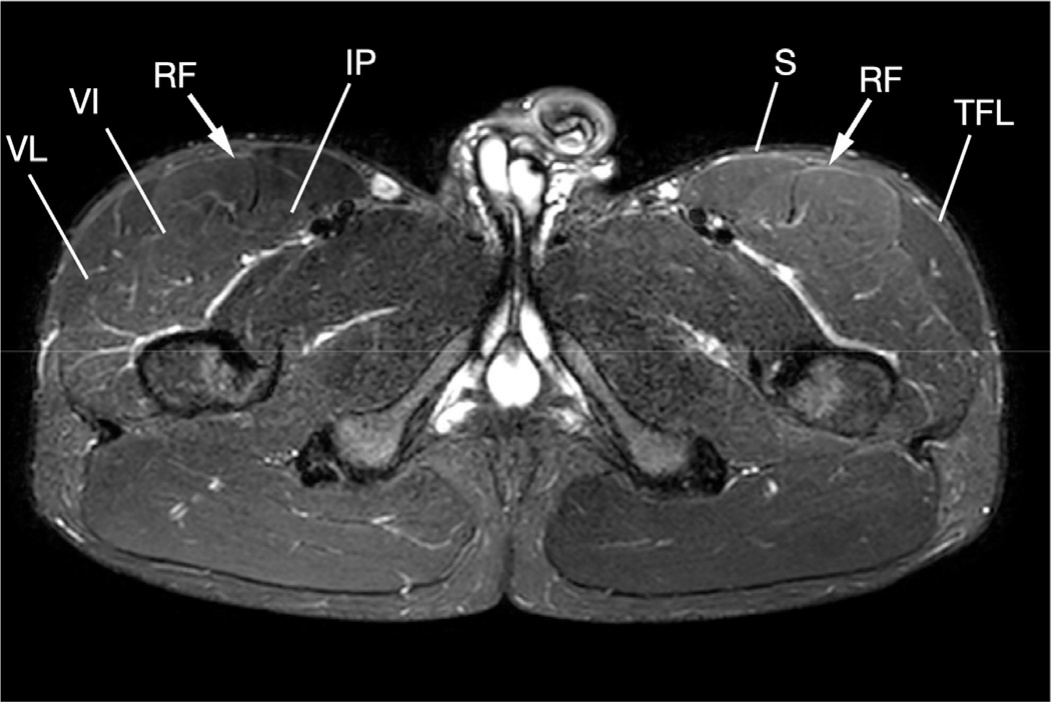
Axial STIR image of the proximal thighs (1 year later, postselective fasciotomy) demonstrates markedly reduced but persistent symmetrical muscular edema involving the bilateral rectus femoris (RF, arrowed) and vastus muscles (VI = vastus intermedius, VL = vastus lateralis), limited to the proximal thighs, with sparing of the hamstrings, adductors, sartorius (S), and tensor fascia lata (TFL) muscles bilaterally.

Due to the nonresolving, recurrent exertional symptoms, the patient was referred to the National Orthopaedic Centre for opinion and subsequent selective fasciotomies of both rectus femoris muscles. This led to improvement of symptoms. While the patient continues to exhibit milder symptoms, appearances on follow-up MR imaging (also performed after 30 minutes of exercise) 1 year later are now not as pronounced (Fig. 2).

## Discussion

Chronic exertional compartment syndrome (CECS) is classically seen in young athletic people. The etiology is not fully understood with several proposals suggesting repetitive microtrauma, infection, decreased compartmental elasticity or eosinophilic fasciitis as causes [2], [3], [4]. Proposed pathophysiology involves increased intramuscular pressures leading to reduced perfusion and subsequent ischemic pain. This manifests as recurrent, exercise-induced muscle pain. It has been suggested that old injuries can cause scar formation and/or adhesions which can lead to this decreased circulation and ischemia [1]. Anabolic steroids and creatine use have also been linked with CECS due to increased muscle volume [5].

The typical clinical presentation of CECS involves the emergence of pain in a specific muscle compartment upon initiating a particular physical activity. This pain intensifies as the activity continues and tends to diminish and resolve during periods of rest. Clinical examination might reveal diffuse tenderness over the affected compartment, particularly after exercise triggers symptoms.

In order to diagnose CECS, the gold standard involves monitoring intracompartmental pressure. This is achieved by inserting a needle into the affected compartment and using a transducer to measure the pressure before and immediately after exercise. Pressure criteria for diagnosis have been proposed but not universally adopted [6]. For additional diagnostic purposes, postactivity MR imaging can also be useful. In cases of CECS, this imaging typically shows a more significant and often delayed increase in T2 signal intensity within the affected compartment following exercise, unlike asymptomatic individuals [7]. Non-CECS subjects typically exhibit peak muscle T2 signal intensity during exercise, which tends to normalize within 15 minutes post exercise. Conversely, individuals with CECS display the highest T2 signal intensity during the initial recovery phase after exercise, and abnormal T2 signal intensity persists for several minutes. Ultimately however, the diagnosis is generally made with a combination of history, physical examination, measurement of compartment pressures and/or MR imaging.

Multiple muscle compartments in the upper and lower limbs can be impacted by CECS, with the lower leg being the most commonly affected [8]. Certain patterns can be seen such as forearm muscles in rowers/gymnasts [9]. But proximal limb muscle involvement is less common. Many cases exist of acute compartment syndrome in the anterior thigh [10], [11], [12], [13], [14], [15]. While we found 3 articles on CECS in the posterior thigh [16,17] and a 2-patient case-series of CECS involving the tensor fascia lata [18], there is only a single Finnish article from 1998 that describes CECS of the anterior thigh. This article reports 9 patients over a period of 14 years, all of whom underwent fasciotomy with improvement in symptoms [19]. Four of the 9 patients had MRI scans (prefasciotomy) performed on a low magnetic strength 0.1T MRI, with only 2 of them having positive findings of increased T2 signal in the quadriceps muscles bilaterally. Our case similarly has positive findings on a higher strength 3T scanner and also features postfasciotomy MR imaging. However, unlike our case, no intracompartmental pressure studies were performed in these patients.

In summary, this case describes a rare presentation of CECS in the anterior thigh, along with the pre- and postoperative MR imaging appearances.

## Patient consent

I confirm that written, informed consent for publication of their case was obtained from the patient.
